# Beta-blocker migraine prophylaxis affects the excitability of the visual cortex as revealed by transcranial magnetic stimulation

**DOI:** 10.1007/s10194-011-0401-x

**Published:** 2011-11-17

**Authors:** Marcus Gerwig, L. Niehaus, P. Stude, Z. Katsarava, H. C. Diener

**Affiliations:** 1 Department of Neurology, University of Duisburg-Essen, Hufelandstr. 55, 45147 Essen, Germany; 2 Department of Neurology, Otto-von-Guericke-University, Magdeburg, Germany; 3 Department of Neurology, University Hospital Bergmannsheil, Bochum, Germany

**Keywords:** Cortical excitability, Migraine prophylaxis, Phosphene threshold, Transcranial magnetic stimulation

## Abstract

The objective of this study is to assess effects of beta-blocker migraine prophylaxis on cortical excitability determined by transcranial magnetic stimulation (TMS). Phosphene and motor thresholds (PT, MT) were investigated in 29 patients with migraine, in 15 of them prior to and following preventive medication with metoprolol and in 14 patients without prophylaxis. Following prophylaxis headache frequency significantly decreased (*p* = 0.005) and mean PT were significantly increased (51.5 ± 7.5 vs. 63.6 ± 8.4%) compared to patients without preventive treatment (53.7 ± 5.3 vs. 52.3 ± 6.3%; *p* = 0.040). Mean MT did not significantly differ either between groups or due to treatment. In the group of all patients, a significant inverse correlation between headache frequency and the level of PT was found (*R* = −0.629; *p* < 0.01). There was, however, no significant correlation in the subgroups of patients. We conclude that (a) clinical efficacy of beta-blocker treatment in migraine could be (at least partly) linked to its ability to modulate the excitability of the visual cortex and (b) the PT determined by TMS appears suitable to assess the effects of prophylaxis on cortical excitability in the individual patient. This may be useful in clinical trials investigating migraine preventive drugs.

## Introduction

Migraine is one of the most frequent neurological disorders and many patients require not only acute attack treatment but also prophylactic medication. However, the exact mechanisms of preventive anti-migraine treatment are still unclear and may be multiple. Among various drugs with different pharmacological properties used for prophylaxis of migraine episodes, beta-blockers have been established as highly effective agents. Propranolol and metoprolol are well-documented substances, for review see [1, 2]. There is increasing evidence in recent years that brainstem as well as cortical dysfunction is basically involved in the complex pathophysiology of migraine. Modified neuronal excitability may be one explanation for preventive pharmacological effects. Electrophysiological and functional imaging studies disclosed abnormalities of cortical information processing [3–5] and of intracortical excitability in migraine, predominantly within the visual cortex, for review see [6]. As a common finding of altered cortical excitability increased mean amplitudes of the contingent negative variation (CNV) and lack of habituation of amplitudes of visual evoked potentials in the interictal state have been reported in migraine patients [7–12]. Normalized amplitudes and habituation of evoked potentials following treatment with beta-blockers [13–15] and valproic acid [16] provide some evidence that central neuronal excitability changes in migraine patients due to prophylactic medication.

As a different approach to explore noninvasively the excitability of motor and visual cortical areas, transcranial magnetic stimulation (TMS) has been established in patients with migraine. Findings on thresholds for eliciting phosphenes (phosphene threshold, PT) in migraine patients are controversial. Most studies reported decreased PT suggesting a higher excitability of the visual cortex between attacks [17–22], but opposite findings (increased PT, reduced excitability) have been reported by some authors [11, 23]. Using TMS an increase of mean PT in migraine patients was found in few uncontrolled trials following treatment with valproate [19], topiramate [24] and levetiracetam [25].

The aim of the present study was to determine whether motor and phosphene thresholds may change due to preventive treatment with the beta-blocker metoprolol in patients with migraine with and without aura. To assess the effect of treatment on cortical excitability, thresholds before and after medication were compared with findings in a group of non-treated migraine patients.

## Methods

All patients were recruited from the headache outpatient center of the neurological department of the University of Duisburg-Essen. The study was approved by the local ethics committee and informed consent was obtained from all subjects.

### Patients

Clinical characteristics of the patients are summarized in Table 1. A total of 29 right-handed outpatients who met the criteria for migraine [26] and who were free from any preventive medication during the last 6 months were investigated. Patients were allowed to use acute headache medication. Inclusion criteria were migraine with aura (MwA) or without aura (MwoA), age 18–65 years and duration of illness >6 months. Patients with chronic migraine or overuse of analgetics and other acute migraine or pain medication were excluded from the study. Further exclusion criteria were epilepsy, pregnancy, mental disorder or substance abuse. The preventively treated group consisted of unselected patients with current migraine attacks of at least two per month. In these15 patients (2 male, 13 female; 6 with MwA, 9 with MwoA; mean attack frequency 3.33 ± 0.97 per month; mean age 37.3 ± 11.5 years, range 18–60 years; mean disease duration 15.3 ± 12.9 years) preventive migraine therapy with metoprolol was initiated by the headache outpatient center. The patients were compared with a group of migraine patients who did not receive preventive treatment by the headache outpatient center consisting of 14 age- and sex-matched patients with lower headache frequencies up to two per month (2 male, 12 female; 7 with MwA, 7 with MwoA; mean attack frequency 1.43 ± 0.90 per month; mean age 37.4 ± 13.8 years, range 21–64 years; mean disease duration 12.2 ± 8.9 years).

**Table 1:** Clinical characteristics of migraine patients

Clinical characteristics	Patients treated with beta-blocker	Patients without prophylaxis
*N*	15	14
MwA/MwoA	6/9	7/7
Male/female	2/13	2/12
Mean age (years)	37.3 ± 11.5	37.4 ± 13.8
Age range (years)	18–60	21–64
Mean disease duration (years)	15.3 ± 12.9	12.2 ± 8.9
Mean attack frequency/month	3.33 ± 0.97	1.43 ± 0.90

Group mean values are expressed ± standard deviation (SD)

*MwA* migraine with aura, *MwoA* migraine without aura

### Study design

Metoprolol was started with 25 mg per day and increased by 25–50 mg per week up to the target dosage of 100 mg per day, which was continued for 8 weeks. In each patient a headache diary was used to assess headache frequency at baseline and on follow-up evaluation. All electrophysiological studies were performed at least 3 days before or after a migraine attack or acute migraine treatment. This was confirmed by a phone call 1 week after the examination. In female patients the time of measurement was distributed randomly throughout both phases of the menstrual cycle. Moreover, the anti-migraine drugs and doses taken for attack treatment were recorded. Headache frequency (number of attacks per month) at baseline was calculated within the last 6 months retrospectively. Baseline measurements were done before start of the prophylactic medication in patients of the treatment group. The follow-up evaluation was performed 8 weeks after the start of treatment. In the same manner TMS measurements were performed twice in patients without preventive medication.

### Magnetic stimulation

TMS procedures have been described in detail previously [22] and are repeated briefly. TMS was performed using a Medtronic Dantec MagPro stimulator (Dantec, Skovlunde, Denmark) and an eight-shaped coil (2 × 10 coil windings, outer diameter 10 cm) and by one investigator (M. G.) who was blinded to the treatment status of the patients.

### Motor threshold

Motor thresholds were determined while the coil was placed over the hand associated primary motor cortex of the right hemisphere, the handle was directed posteriorly. First, stimuli were applied with suprathreshold intensity and the coil was moved in steps of 1 cm to determine the optimal scalp position for producing motor evoked potentials (MEP) of maximal amplitude (lowest threshold) in the contralateral target hand muscle. Stimulus intensities then were reduced in steps of 2% of maximal stimulator output. MEP were recorded with bipolar surface electrodes attached over the first dorsal interosseus muscle. The electromyographic signals were amplified with band pass filtering between 20 Hz and 3 kHz and recorded with a personal computer using an analogue digital converter (CED 1401 plus Interface, recording frequency 5,000/sec per channel) and a data registration program (SigAvg; CED, Cambridge, UK). Threshold for eliciting MEP was defined as the lowest stimulus intensity (% of maximal stimulator output) capable of eliciting at least five MEP with an amplitude of at least 100 μV in a relaxed hand muscle in a series of ten consecutive trials of TMS [27].

### Phosphene threshold

For elicitation of phosphenes the coil was centered over the occipital skull with the handgrip pointing horizontally into lateral direction [28]. The MagPro stimulator provides pulses (biphasic pulses; duration: 340 μs) with identical stimulus intensities through the same coil. The subjects were blindfolded and wore a swimmer cap with a surface grid system of 1 × 1 cm intersections parallel to the medio-sagittal line and the interaural line. This was used to enable the exact repositioning of the coil.

The subjects were asked to report every bright or colored visual perception during TMS. Due to a previous methodological paper the stimulation procedure started with suprathreshold TMS pulses over the right visual cortex to provide an experience of phosphene perception to the subjects [29]. The coil was moved in steps of 1 cm in mediolateral and craniocaudal direction to identify the optimal position in which brief flashes or white patches of light were consistently reported foveally or within the left visual hemifield. At this point, phosphene thresholds were determined in each subject. For this purpose TMS was initially applied with a stimulus intensity of 20% of maximal stimulator output and further increased in steps of 5% until phosphenes were reported. Then, the threshold was fine-tuned by varying the stimulus intensity in steps of 2%. To avoid order effects, we randomized the direction (increasing/decreasing) in which the stimulus intensity was changed. PT was defined as the minimal stimulus intensity, at which the subjects reported phosphenes in at least five out of ten stimulations at a given coil position. The intertrial intervals were at least 10 s. To avoid changes of visual cortex excitability due to a prolonged sensory deprivation, the examination was interrupted every 10–15 min and light exposure was provided to the subjects [30].

### Data analysis

SPSS software (Version 14.0) was used for statistical evaluation. Analysis of variance with repeated measures (ANOVA) was performed to compare PT and MT as well as migraine frequency between the first and second assessment, i.e. time as within subject factor and groups that is prophylactic treatment versus no treatment as between subjects factor and to calculate the effect of treatment on thresholds (interaction effect). Level of significance with *p* < 0.05 was accepted. Statistical evaluation of headache frequency was based on time series analysis of variance of groups, i.e. preventively treated patients and non-treated patients. Response to treatment was defined as reduction of at least 50% of migraine episodes per month. The Spearman Pearson Product Moment Correlation coefficient was used to analyze the relation between headache frequency and PT and changes of headache frequency and PT in migraine patients with and without preventive treatment.

## Results

### Clinical efficacy

Comparing the individual differences of headache frequencies at the first and second testing, in 11 out of the 15 treated patients (73.3%) migraine episodes were reduced ≥50% at second testing. The mean frequency of migraine attacks significantly decreased following beta-blocker treatment (3.33 ± 0.97 vs. 1.47 ± 1.26 per month), but was lower and remained nearly unchanged in patients without prophylaxis (1.43 ± 0.90 vs. 1.30 ± 0.73 per month). Comparison of change of headache frequencies between groups showed significant time (*F*_1,27_ = 32.6; *p* < 0.001) and time by group effects (*F*_1,27_ = 24.8; *p* < 0.001), the group effect was also significant (*F*_1,27_ = 9.5; *p* = 0.005).

### Motor thresholds

MT ranged from 30 to 48% of maximal stimulator output in treated and from 32 to 55% in the non-treated group. Comparison of mean MT at baseline and follow up between groups (38.0 ± 5.8 vs. 38.9 ± 4.4% in treated and 39.3 ± 5.9 vs. 40.7 ± 6.1% in non-treated patients) did not reveal a significant time by group effect (*F*_1,27_ = 0.30; *p* > 0.5), also the group effect (*F*_1,27_ = 0.56; *p* = 0.45) was not significant.

### Phosphene thresholds

**Fig. 1:**
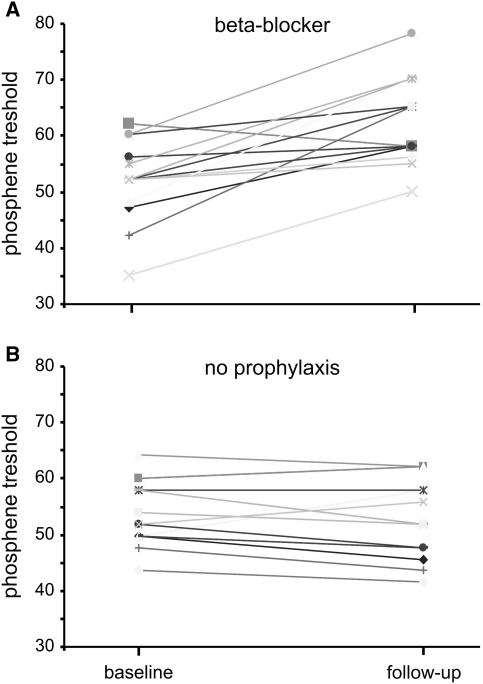
Individual phosphene thresholds (% of the maximal stimulator output) in migraine patients determined by single-pulse TMS at baseline and following beta-blocker treatment (**a**) and in patients without preventive treatment (**b**)

**Fig. 2:**
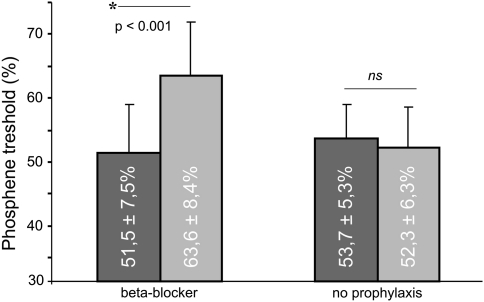
Mean phosphene thresholds ± SE at baseline (*dark blue columns*) and follow-up (*light blue columns*) in preventively treated patients compared to patients without migraine prophylaxis

**Fig. 3:**
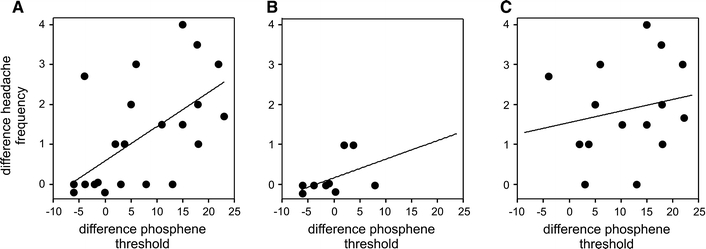
Correlation between changes of attack frequencies per month and differences of phosphene thresholds in all patients (**a**), patients without migraine prophylaxis (**b**) and patients with beta-blocker treatment (**c**). Note that *several dots* in (**a**) and (**b**) represent two or more subjects

Following occipital stimulation phosphenes were reported by all subjects, located foveally or within the lower quadrants of the visual field. Optimal stimulation sites to elicit phosphenes lay 1–5 cm above the inion. There were no adverse effects related to TMS in any of the participants. Individual phosphene thresholds determined by single-pulse magnetic stimulation are shown in each subject with and without preventive treatment at baseline and follow-up measurement (Fig. 1). At second testing thresholds were enhanced in 14 of the treated patients. However, in the 11 patients who clinically responded to treatment PT increase ranged from 4 to 23% of maximum stimulator output, in one subject PT was slightly decreased following treatment. Conversely, in two of the four non-responding patients PT were enhanced at second testing. This was the case in three of the non-treated patients, in five of them thresholds were decreased at second testing. Mean PT was not different between groups at baseline, but was increased following preventive treatment in contrast to the non-treated patients (51.5 ± 7.5 vs. 63.6 ± 8.4% and 53.7 ± 5.3 vs. 52.3 ± 6.3%; Fig. 2). ANOVA with repeated measures with PT as dependent variable, time as within subjects factor and treatment status as between subjects factor revealed a significant time by group effect (*F*_1,27_ = 27.9; *p* < 0.001) and a significant group effect (*F*_1,27_ = 4.7; *p* = 0.040) indicating that mean PT were significantly enhanced following treatment. No significant difference was found comparing mean PT at baseline and PT enhancement in MwA-and MwoA-patients of the treated group. In the group of all subjects headache frequency was inversely and significantly correlated with the level of PT (*R* = −0.629, *p* < 0.01; Fig. 3a). Correlation was not significant in subgroups of patients either in treated patients (*R* = −0.220, *p* = 0.43) or in the non-treated patients (*R* = −0.491, *p* = 0.075). Furthermore, analysis of PT change versus clinical change did not reveal any significant correlation either in patients responding to treatment (*R* = −0.70, *p* = 0.837) or in non-responders (*R* = 0.219, *p* = 0.781).

## Discussion

In patients of the present study the frequency of migraine attacks was inversely correlated with the level of phosphene thresholds. In a subgroup of patients, treatment with metoprolol resulted in a reduction of mean attack frequency and an increase of mean PT to a level observed previously in a group of healthy controls [22]. In these patients the headache frequency was higher than in a control group of patients without migraine prophylaxis. The findings suggest that preventive treatment with a beta-blocker in migraine may reduce the excitability of the visual cortex. However, clinical efficacy was not significantly correlated with enhancement of mean PT.

The significant decrease of headache frequency in the present patients following treatment is consistent with the good evidence of effective migraine prevention by beta-blocking agents [31–33]. The underlying mechanisms of the prophylactic efficacy of substances used in migraine prevention are a matter of ongoing discussion. One explanation centers on a modified central neuronal excitability due to treatment. This is supported by the present findings of enhanced mean PT following prophylactic medication. Comparable to the corticomotor threshold, phosphene thresholds have been shown to represent a reliable parameter of visual cortex excitability in healthy subjects and to be stable across repeated measurements [34–36]. Mean PT remained unchanged in the present patients without preventive treatment. Interestingly, values tended to be lower at second testing possibly because the subjects were more experienced in the recognition of phosphenes.

In recent years many studies investigated electrophysiological parameters in migraine [6]. Following beta-blocker treatment altered amplitudes and habituation of the contingent negative variation [13, 37] as well as amplitudes and latencies of pattern reversal visual evoked potentials [14] were reported to normalize. Also in the present patients mean PT increased to values corresponding to that in healthy controls as assessed in a previous trial. Consistent with earlier findings the data suggest that modified central excitability may be one factor of prophylactic efficacy of beta-blocker treatment in migraine and that dysfunction of cortical excitability is at least associated with mechanisms underlying the pathophysiology of migraine.

However, lack of significant correlation of PT enhancement with clinical improvement shows that PT related changes of cortical excitability do not appear to predict per se preventive efficacy. This is supported by findings in individual patients. Two out of four patients, although not responding to treatment, showed higher PT at second testing. Conversely, single patients out of the clinical responders showed only slight or no increase of PT following treatment. Although most migraineurs may be characterized by modified cortical excitability, it therefore cannot be definitely assessed that it is one of the causes of the disease and not just an epiphenomenon strictly linked to the disease. Moreover, as clinical response may represent a placebo (in common clinical trials about 30%) rather than a biological effect, attribution of the clinical efficacy to the treatment or PT change on a case-by-case basis remains uncertain.

The current data are consistent with a previous, uncontrolled study using valproate, also known to be effective in migraine prevention [19]. Following medication ascent of low mean PT was observed, however, only in MwA patients and correlation of PT enhancement with clinical efficacy tended to significance. Two further uncontrolled TMS studies were conducted using other antiepileptic drugs for migraine prophylaxis. In an open label trial with levetiracetam change of headache frequency and change of PT were negatively correlated at the 10% but not at the 5% significance level [25]. Mean PT were also increased following preventive treatment with topiramate in patients with migraine without aura [24]. Surprisingly, an inverse correlation was reported between decrease of migraine frequency and increase of mean PT. In the present study, however, no significant difference of mean PT at baseline and PT enhancement due to treatment was observed comparing MwA- and MwoA-patients. Findings of previous studies and the present data suggest that migraine prophylaxis with different centrally acting substances may be more complex and cannot only be explained by modification of cortical excitability. A non-linear relation between migraine activity and PT in patients with frequent migraine has also been discussed [24].

Animal data provide strong evidence for the implication of cortical spreading depression in migraine pathophysiology. In rats it has been shown that chronic treatment with different migraine prophylactic drugs suppresses cortical spreading depression in a duration-dependent manner and increases the electrical stimulation threshold [38]. Since increased central noradrenergic activity may be a basic phenomenon in the pathophysiology of migraine, findings of the present and earlier studies following beta-blocker treatment suggest that one mechanism of migraine prophylaxis may be to act on cortical hyperexcitability.

In the present patients mean MT did not change due to preventive treatment. Studies evaluating MT over the primary motor cortex in patients with migraine revealed controversial results [11, 39–41], no significant changes of MT in migraine were reported more recently [34, 42]. Following treatment with topiramate mean MT was enhanced, again there was no correlation with clinical effects [24]. However, even if report of excitability dysfunction in migraine are controversial concerning the MT assessment, motor cortex plasticity has been found to be consistently altered (short term plasticity and homeostatic plasticity) in the direction of increased responsivity in migraine [43, 44]. Indeed changes in plasticity more than pure hyper- or hypoexcitability are likely more specific for migraine pathophysiology [43, 44]. Moreover, modulation of such mechanisms by migraine prophylaxis has been shown in the visual and motor cortex [43–45].

In conclusion the present findings suggest that effects of beta-blockers in migraine prevention are, at least in part, related to influences on the excitability of the visual cortex. Due to treatment decreased mean PT were restored to a level corresponding to that in healthy controls as shown in a previous study. This was not observed in all subjects who responded to treatment suggesting that phosphene thresholds elicited by occipital TMS appear suitable to measure effects of prophylaxis on cortical excitability only in individual patients.

There are several limitations of the study. The baseline PT tests gave similar results in both groups, who were different for headache frequency. It might be useful to investigate patients with comparable attack frequencies at baseline. Moreover, there was a higher responder rate compared to clinical trials which may be due to the relatively small sample size and may therefore not reflect data of larger clinical trials. Finally, the design was not randomized and there was no placebo control.

Lack of significant correlation in treated patients of the present study, however, suggests that clinical efficacy of migraine prevention may in addition depend on effects distinct from cortical excitability [24]. Imaging studies have shown that the rostral brainstem may enhance cortical activation in migraine [46, 47] and effects of migraine preventive drugs on other brain structures are likely. Further studies with larger groups of patients are needed to evaluate whether and to what extent clinical effects of substances used in migraine prevention may be related to cortical excitability and to determine long lasting effects.
